# Complete Nucleotide Sequence of *Klebsiella pneumoniae* Bacteriophage vB_KpnM_KpV477

**DOI:** 10.1128/genomeA.00694-17

**Published:** 2017-09-14

**Authors:** Ekaterina V. Komisarova, Angelina A. Kislichkina, Valentina M. Krasilnikova, Alexander G. Bogun, Nadezhda K. Fursova, Nikolay V. Volozhantsev

**Affiliations:** State Research Center for Applied Microbiology and Biotechnology, Obolensk, Moscow Region, Russia

## Abstract

The double-stranded DNA (dsDNA) bacteriophage vB_KpnM_KpV477, with a broad spectrum of lytic activity against *Klebsiella pneumoniae*, including strains of capsular serotypes K1, K2, and K57, was isolated from a clinical sample. The phage genome comprises 168,272 bp, with a G+C content of 39.3%, and it contains 275 putative coding sequences (CDSs) and 17 tRNAs.

## GENOME ANNOUNCEMENT

*Klebsiella pneumoniae* is the most common clinically important pathogen causing both community-acquired and hospital-acquired infections (1, 2). In recent years, two clinical problems have been associated with *K. pneumoniae*, the spread of multidrug-resistant (MDR) strains (3) and the emergence of hypervirulent (hypermucoviscous) variants belonging mainly to K-1, K-2, K-57, and some other capsule serotypes (4). Lytic bacteriophages are considered the most accessible and acceptable alternative (additive) to antibiotics against *K. pneumoniae* infections (5–7).

In this study, we report the genome sequence of the *Klebsiella* bacteriophage vB_KpnM_KpV477 (here, KpV477) isolated from a clinical sample obtained from the Burdenko Neurosurgery Institute (Moscow, Russia) and deposited in the State Collection of Pathogenic Microorganisms and Cell Cultures SCPM-Obolensk (accession no. Ph-112). The phage was shown to lyse with plaque formation 54 of 246 *K. pneumoniae* strains of capsular types K-1, K-2, K-57, and some others, including MDR strains.

Phage KpV477 was propagated on *K. pneumoniae* strain KPB463 (SCPM-Obolensk accession no. В-7848), and its DNA was sequenced using the Ion Torrent PGM platform (Life Technologies, Inc., USA). The resultant 21,335 reads, with an average length of 245 bases and coverage equal to 31-fold, were successfully assembled into a single contig using Newbler 2.9. The correctness of assembly was checked using the SeqMan NGen software (DNAStar, Madison, WI, USA).

The whole genome of phage KpV477 was presented as a linear double-stranded DNA, with a length of 168,272 bp and a G+C content of 39.3%. Coding sequences (CDSs) within the KpV477 genome were allocated using the software tools GeneMarkS (8) and Prodigal (9). It was shown that the phage KpV477 genome has 275 CDSs on both strands of the DNA that are presumably organized into 66 transcriptional units, as determined by FgenesB (Softberry). Out of 275 CDSs, 263 potential genes have with the ATG initiation codon. Eight CDSs start with GTG, and another four sequences start with TTG. Prodigal (9) analysis showed that 259 CDSs are preceded by consensus sequences of potential ribosome binding sites. A gene cluster encoding 17 tRNAs for 14 amino acids (Arg, Asn, Asp, Gln, Gly, His, Ile, Leu, Lys, Met, Pro, Thr, Trp, and Tyr) and Pseudo-TGA were identified in the phage genome using tRNAscan-SE (10).

Putative functions were assigned to 111 of 275 CDSs predicted products based on similarity with known proteins and identification of conserved domains at the National Center for Biotechnology Information (NCBI) databases. Genes related to lysogeny, such as integrases, repressors, and antirepressors, expressed during the prophage stage, were not identified in the KpV477 genome. These data, as well as the results of a one-step growth experiment and analysis using the Phage Classification Tool Set (11), indicate that KpV477 is a lytic bacteriophage.

The BLASTn (12) results showed that the phage KpV477 genome exhibits high similarity (94 to 96%) with *Klebsiella* phages JD18 (GenBank accession no. KT239446) and PKO111 (GenBank accession no. KR269720) belonging to the newly formed genus *JD18virus* (subfamily* Tevenvirinae*, family *Myoviridae*). The collinearity of KpV477, JD18, and PKO111 genomes was confirmed by progressiveMauve analysis (13). As determined by using the BLAST algorithms, only 12 of 275 KpV477 phage genes are not identical to those of JD18 or PKO111. Nine of these genes encode hypothetical proteins with unknown function, two genes encode putative HNH homing endonucleases, and one gene contains a conserved GIY-YIG nuclease domain.

### Accession number(s).

The complete genome of the bacteriophage vB_KpnM_KpV477 was deposited in GenBank under the accession no. KX258185.
